# Phytochemical Composition of *Combretum molle* (R. Br. ex G. Don.) Engl. & Diels Leaf and Stem Extracts

**DOI:** 10.3390/plants12081702

**Published:** 2023-04-19

**Authors:** Myuri Parusnath, Yougasphree Naidoo, Moganavelli Singh, Hail Rihan, Yaser Hassan Dewir

**Affiliations:** 1 School of Life Sciences, University of KwaZulu-Natal, Westville Campus, Private Bag X54001, Durban 4000, South Africa; 2 School of Biological and Marine Sciences, Faculty of Science and Engineering, University of Plymouth, Drake Circus PL4 8AA, UK; 3 Phytome Life Sciences, Launceston PL15 7AB, UK; 4 Plant Production Department, College of Food and Agriculture Sciences, King Saud University, Riyadh 11451, Saudi Arabia

**Keywords:** medicinal plants, phytochemical screening, Fourier transform infrared spectroscopy, energy-dispersive X-ray microanalyses, fluorescence microscopy

## Abstract

The demand for medicinal plants is on a rise due to their affordability, accessibility and relatively non-toxic nature. *Combretum molle* (Combretaceae) is used in African traditional medicine to treat a number of diseases. This study aimed to screen the phytochemical composition of the hexane, chloroform and methanol extracts of *C*. *molle* leaves and stems using qualitative phytochemical screening. Additionally, the study aimed to identify the functional phytochemical groups, determine the elemental composition and provide a fluorescence characterization of the powdered leaves and stems by performing Fourier transform infrared spectroscopy (FTIR), energy-dispersive X-ray (EDX) microanalyses and fluorescence microscopy. Phytochemical screening revealed the presence of alkaloids, flavonoids, phenolic compounds, polyphenols, terpenoids, tannins, coumarins, saponins, phytosterols, gums, mucilage, carbohydrates, amino acids and proteins within all leaf and stem extracts. Lipids and fixed oils were additionally present within the methanol extracts. FTIR demonstrated significant peaks in absorption frequency in the leaf at wavelengths of 3283.18, 2917.81, 1617.72, 1318.83, 1233.97, 1032.32 and 521.38 cm^−1^, and in the stem at 3318.91, 1619.25, 1317.13, 1032.68, 780.86 and 516.39 cm^−1^. These corresponded to the functional groups of chemical compounds including alcohols, phenols, primary amines, alkyl halides, alkanes and alkyl aryl ethers, corroborating the presence of the detected phytochemicals within the plant. EDX microanalyses showed the elemental composition of the powdered leaves (68.44% C, 26.72% O, 1.87% Ca, 0.96% Cl, 0.93% Mg, 0.71% K, 0.13% Na, 0.12 % Mn and 0.10% Rb) and stems (54.92% C, 42.86% O, 1.7% Ca, 0.43% Mg and 0.09% Mn). Fluorescence microscopy provided a characteristic evaluation of the plant in its powdered form and revealed distinct colour changes in the material when treated with various reagents and viewed under ultraviolet light. In conclusion, the phytochemical constituents of the leaves and stems of *C*. *molle* confirm the suitability of this species for use in traditional medicine. The findings from this study suggest the need to validate the use of *C*. *molle* in the development of modern medicines.

## 1. Introduction

The use of herbal medicine has been prevalent for thousands of years [1], and even today, people in disadvantaged communities rely on plant-based treatments due to their affordability, accessibility, and cultural acceptability [2]. Modern medicines may have adverse side effects and low potencies [3,4], making it important to assess alternative medicinal sources. People in the African continent in large part rely on herbal practices, with nearly 27 million South Africans utilizing herbal medicine as a form of primary healthcare [5,6]. Plants contain a plethora of bioactive phytocompounds that can assist in the symptomatic management of disease and combat pathogenic resistance [4,7,8]. These phytocompounds can be categorized into primary and secondary metabolites [9,10]. While primary metabolites are required for the major life processes of a plant and are involved in the biosynthesis of macromolecules (e.g., proteins, nucleic acids, carbohydrates and lipids), secondary metabolites are not essential for a plant’s existence and instead offer protection from herbivory, insects and pathogenic organisms [9]. Phytocompounds in general produce antibacterial, antifungal and antioxidant effects, thus rationalizing the use of plant extracts in disease treatment [10]. Many species from the pantropical family, Combretaceae R. Br., specifically from the genus *Combretum* Loefl., are exploited in African traditional medicine [11]. In particular, *Combretum molle* (R. Br. ex G. Don) Engl. & Diels (velvet bushwillow), a tree prevalent in southern African countries [12], is widely used in the traditional medicinal treatment of numerous diseases. The plant is prevalent in and native to southern Africa and is predominantly located in forest, woodland, and savannah biomes [12,13]. *Combretum molle* has a rounded, dense crown with low hanging branches [14]. The young stems may be characterized as hairy; the leaves have an opposite arrangement, and are simple, with an entire margin and a tapered or rounded apex [14,15]. The leaves bear combretaceous hairs, creating a velvety texture [16]. Various parts of the plant including the leaves and stems are used to treat illnesses such as leprosy, fever, wounds, oedema/dropsy, chest disorders, convulsions, abdominal disorders, angina and HIV/AIDS [11]. The plant’s traditional use may be ascribed to the curative properties of the plant’s bioactive compounds [17].

Several phytochemical studies have been performed on extracts of *C*. *molle*. A triterpene acid saponin, mollic acid glucoside, was previously isolated from the leaves of *C*. *molle* and has been reported to have antifungal [18], anti-inflammatory [19], analgesic, cardioprotective and cardiovascular [20,21] properties. In addition, triterpenoids such as combregenin, arjunglucoside I and II and combreglucoside isolated from the plant’s stembark reportedly possess anti-inflammatory effects [19]. Moreover, phenolic compounds including tannins such as punicalagin found within extracts of *C*. *molle* were reported to exhibit antioxidant, antimalarial [20] and anti-HIV [22] effects. These findings partially confirm the suitability of this plant for use in traditional medicinal practices. The effectiveness of medicinal plants in disease treatment is dependent on the quality and quantity of the bioactive compounds present within the plant [23]. However, the adulteration or substitution of plant material with non-medicinal species or other material is a possibility [24]. Adulteration may occur deliberately to increase the weight of the product and to decrease costs but may also occur unintentionally as a result of species misidentification [24]. In herbal treatment, plant material is primarily crushed into fine powders, resulting in a loss of a species’ characteristic morphological identity [24]. Therefore, to circumvent the issue of adulteration, it is imperative to conduct pharmacognostic studies on medicinal plant species to authenticate the material. In order for the validation of medicinal plants in modern applications, quality control and the use of standardized material must be ensured.

Prior to the validation of medicinal plants in modern drug development, it is imperative to assess the therapeutic properties and bioactive compounds within the plants’ extracts, especially as the demand and consumption of herbal medicines increase [5,7]. The occurrence of several bioactive compounds within *C*. *molle* supports the need for further research on the plant prior to expanding its application in drug development. However, despite numerous phytocompounds having been isolated from leaf extracts of *C*. *molle*, there is a paucity of research on the phytochemistry of the plant’s stem material. This study thus aimed to determine the phytochemical composition of the hexane, chloroform and methanol leaf and stem extracts of *C*. *molle* using qualitative phytochemical screening. It is necessary to perform pharmacognostic evaluations on *C*. *molle* to provide a characterization of the plant in its powdered form. Therefore, this study additionally aimed to identify the functional groups, determine the elemental composition and provide a fluorescence characterization of the plant’s powdered leaves and stems using Fourier transform infrared (FTIR) spectroscopy, energy-dispersive X-ray (EDX) microanalyses and fluorescence microscopical analyses, respectively. To the best of our knowledge, this is the first study detailing the characteristics of the plant’s powdered material via FTIR, EDX and fluorescence microscopy.

## 2. Results

### 2.1. Extract Yield

The percentage yield of the *C*. *molle* leaf and stems extracted in hexane, chloroform and methanol were determined (Table 1). The highest yields were obtained for the methanol extracts, followed by the chloroform and hexane extracts. Overall, the leaf extracts produced higher metabolite yields than the stems.

### 2.2. Preliminary Qualitative Phytochemical Screening

Preliminary qualitative phytochemical screening of the hexane, chloroform and methanol extracts of the *C*. *molle* leaf and stem revealed the presence of various phytocompounds in the plant (Table 2). All of the analyzed extracts presented positive reactions for alkaloids, flavonoids, phenolic compounds, polyphenols, terpenoids, tannins, coumarins, saponins, phytosterols, gums, mucilage, carbohydrates, amino acids and proteins. When tested with Wagner’s and Mayer’s reagents, the extracts produced red-brown and creamy-white precipitates, respectively, confirming the presence of alkaloids. In Wagner’s test, intense results were produced by the methanol extracts. The presence of an intense yellow precipitate during the lead acetate test suggested a strong presence of flavonoids. Flavonoids were additionally detected in all extracts by the alkaline and acid hydrolysis tests, which resulted in the formation of a yellow-to-clear solution, and an intense yellow solution, respectively. The intense yellow colouration produced from acid hydrolysis indicated the presence of flavones and flavanols, specifically. The ferric chloride test presented positive reactions for phenolic compounds, polyphenols and tannins. Phenolic compounds and tannins were confirmed by the formation of a dark green-brown to black solution, whereas polyphenols were confirmed by a green solution. The lead acetate test additionally produced positive reactions for phenolic compounds and tannins by a white precipitate. Tannins were further detected in all the extracts by positive reactions in the gelatin test, shown by the formation of a white precipitate. The Salkowski’s and Liebermann–Burchard tests yielded positive results for terpenoids and phytosterols in all six extracts. Terpenoids were confirmed by a dark green solution with a red-brown colouration at the interface, and phytosterols were confirmed by a red-brown colouration with a green, fluorescent ring, accompanied with a colour change from violet to blue, and thereafter to green. The sodium hydroxide test resulted in positive results for coumarins in all extracts by the formation of a yellow precipitate. Saponins were detected in all extracts via the foam test, as indicated by the formation of a foamy layer. However, the hexane and chloroform extracts presented negative reactions for saponins when analyzed using the olive oil test. Positive results were obtained for the methanol extracts where a soluble emulsion was formed after shaking vigorously. The hexane and chloroform extracts produced negative results for lipids and fixed oils, while the methanol extracts exhibited intense positive results by producing oil stains on the filter paper during the spot test. While the hexane extracts produced a positive reaction for gums and mucilage by the formation of a cloudy-white precipitate during the precipitation test, no reactions were observed in the chloroform and methanol extracts, indicating the absence of gums and mucilage in these extracts. The ruthenium red test, however, yielded positive results for gums and mucilage in all extracts, as confirmed by the formation of a pink solution. Carbohydrates were detected in all extracts by Molisch’s test, where a purple-violet ring formed upon the addition of sulphuric acid, as well as by Fehling’s test, where a red precipitate was observed. Amino acids and proteins were additionally detected in all extracts by positive reactions from the ninhydrin and biuret tests, which produced a purple colour in the solutions. All extracts presented negative reactions for resins via the acetone test, as well as for glycosides via the Keller–Killani test, where no changes or reactions were observed.

### 2.3. FTIR Spectroscopy

**Figure 1:**
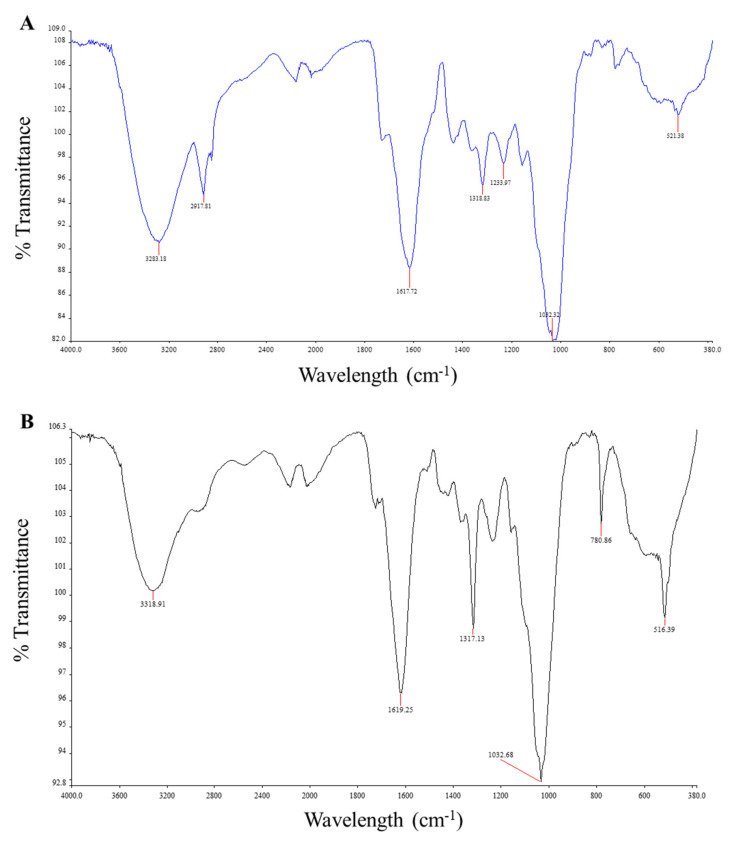
FTIR spectra of powdered (**A**) leaf and (**B**) stem samples of *Combretum molle*.

FTIR analyses confirmed the presence of phytocompounds by identifying the functional groups present within the powdered leaf and stem samples of *C*. *molle*. The FTIR spectra demonstrated significant peaks in absorption frequency at 3283.18, 2917.81, 1617.72, 1318.83, 1233.97, 1032.32 and 521.38 cm^−1^ for the powdered leaf (Figure 1A), and at 3318.91, 1619.25, 1317.13, 1032.68, 780.86 and 516.39 cm^−1^ for the powdered stem (Figure 1B) samples (Table 3). Using the spectra, the intensity, corresponding functional groups, compound classes and vibration type for each peak in absorption frequency were determined (Table 3). A majority of the detected functional groups were present in both the leaf and stem powdered material. The strong, broad peaks occurring at 3283.18 cm^−1^ in the leaf spectra and at 3318.91 cm^−1^ in the stem spectra corresponded to the O-H (stretching) functional group of alcohols. Medium peaks detected at 1617.72 cm^−1^ and 1619.25 cm^−1^ in the leaf and stem, respectively, corresponded to the N-H (bending) functional groups of primary amines. The occurrence of medium peaks at 1032.32 cm^−1^ in the leaf and 1032.68 cm^−1^ in the stem further signified the presence of primary amines by its C-N (stretching) functional group. Additional medium peaks in the leaf and stem spectra at wavelengths of 1318.83 and 1317.13 cm^−1^, respectively, were indicative of the O-H (bending) group of phenols. The analyses also detected the presence of alkyl halides (halo compounds) in both samples. The spectra produced strong peaks at 521.38 and 516.39 cm^−1^, corresponding to the C-I (stretching) functional group of alkyl halides, for the leaf and stem, respectively, as well as a strong peak corresponding to the C-Cl (stretching) group at 780.76 cm^−1^ in the stem only. In addition, the leaf spectra detected a medium peak at 2917.81 cm^−1^ and a strong peak at 1233.97 cm^−1^, indicative of the C-H (stretching) group of alkanes and the C-O (stretching) group of alkyl aryl ethers, respectively. 

### 2.4. EDX Microanalyses

**Figure 2:**
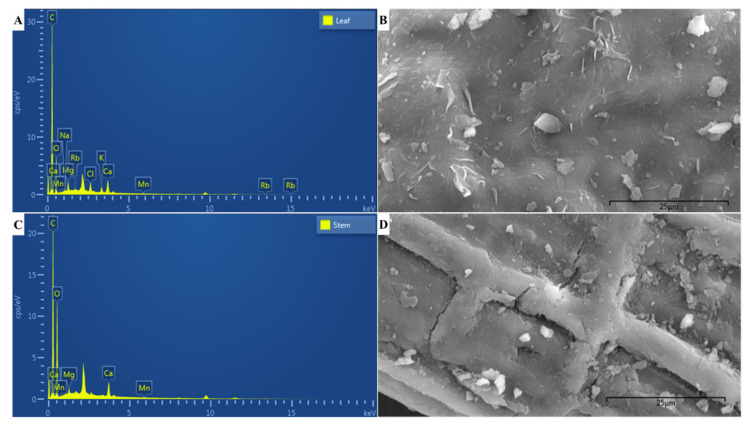
Elemental composition of powdered leaf and stem samples of *Combretum molle*. (**A**) EDX spectrum of powdered leaf; (**B**) SEM micrograph of powdered leaf particles used for EDX microanalyses; (**C**) EDX spectrum of powdered stem; (**D**) SEM micrograph of powdered stem particles used for EDX microanalyses.

EDX microanalyses revealed the elemental composition of the powdered leaf (Figure 2A,B) and stem (Figure 2C,D) samples of *C*. *molle* at a microscopic scale. The EDX spectra (Figure 2A,C) and percent elemental composition (Table 4) revealed that carbon was the most prominent element in both samples, making up 68.44% and 54.92% of the powdered leaf and stem, respectively. This was followed by 26.72% and 42.86% oxygen, and 1.87% and 1.70% calcium, in the leaf and stem, correspondingly. All other elements were detected in minute quantities (<1.00%), with the leaf comprising 0.96% chlorine, 0.93% magnesium, 0.71% potassium, 0.13% sodium, 0.12% manganese and 0.10% rubidium, and the stem containing 0.43% magnesium and 0.09% manganese.

### 2.5. Fluorescence Microanalyses 

**Figure 3:**
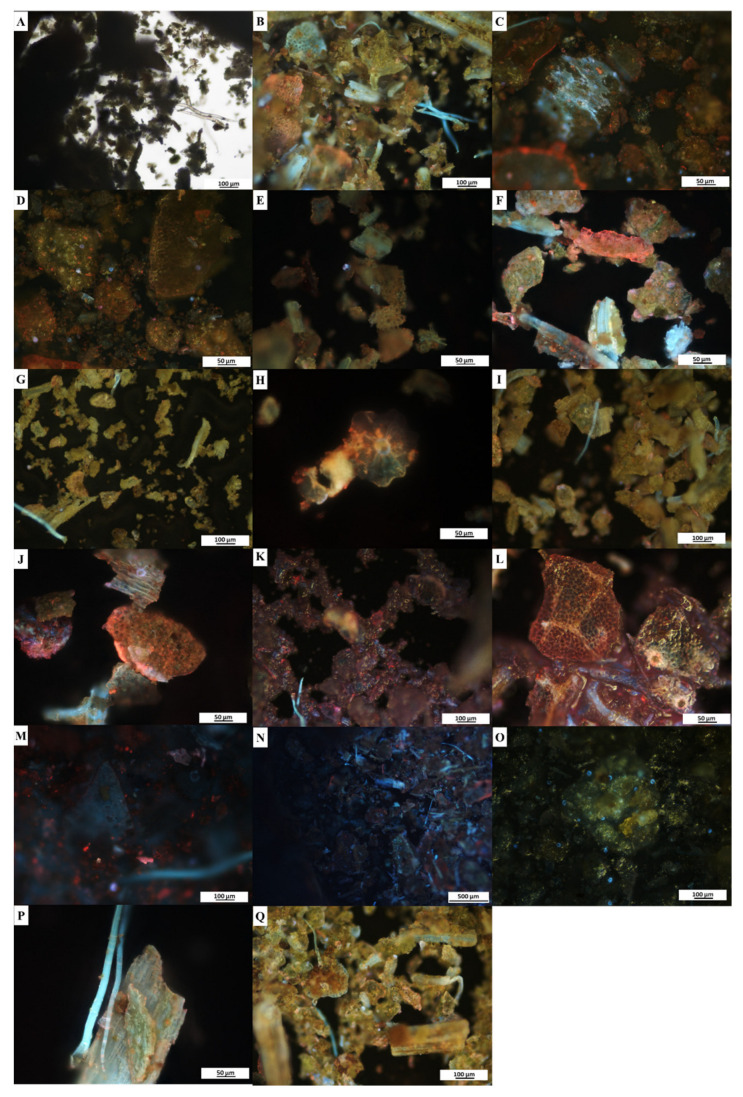
Fluorescence microanalyses of powdered leaf material of *Combretum molle*. Powder only under (**A**) brightfield and (**B**) UV light (control); powder treated with: (**C**,**D**) distilled water; (**E**) hexane; (**F**) chloroform; (**G**,**H**) methanol; (**I**,**J**) ethanol; (**K**,**L**) acetic acid; (**M**) sodium hydroxide; (**N**) sulphuric acid; (**O**) hydrochloric acid; and (**P**,**Q**) acetone under UV light.

**Figure 4:**
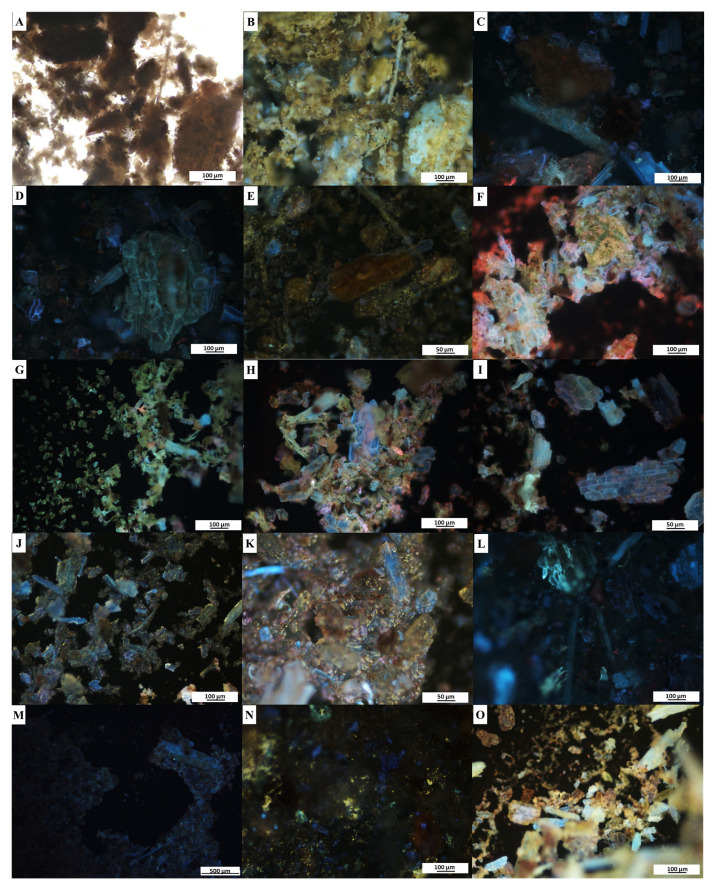
Fluorescence microanalyses of powdered stem material of *Combretum molle*. Powder only under (**A**) brightfield and (**B**) UV light (control); powder treated with: (**C**,**D**) distilled water; (**E**) hexane; (**F**) chloroform; (**G**) methanol; (**H**,**I**) ethanol; (**J**,**K**) acetic acid; (**L**) sodium hydroxide; (**M**) sulphuric acid; (**N**) hydrochloric acid; and (**O**) acetone under UV light.

Fluorescence microanalyses of the powdered leaf and stem samples of *C*. *molle* were conducted to provide a pharmacognostic characterization of the plant material in powdered form. When viewed under brightfield light, the powdered leaf (Figure 3A) and stem (Figure 4A) samples for all treatments appeared green and brown, respectively (Table 5). Under ultraviolet light, all treatments produced a distinct colour change with fluorescence (Table 5, Figure 3B–Q and Figure 4B–O). The colour observations varied between the leaf (Figure 3B–N) and stem (Figure 4B–M) for a majority of the treatments; however, similar changes were observed in the hydrochloric acid (Figure 3O and Figure 4N), acetone (Figure 3P,Q and Figure 4O) treatments. The fluorescence analyses revealed several cellular segments, including the plant’s characteristic peltate glandular scales which fluoresced orange and blue when treated with methanol (Figure 3H). The plant’s non-glandular trichomes (Figure 3B,G,I,K,M,P,Q) fluoresced blue in several treatments, despite variations in reagents.

## 3. Discussion

### 3.1. Yield and Phytochemical Composition of Leaf and Stem Extracts

Medicinal plants contain an abundance of bioactive compounds that effectively manage symptoms and treat disease with little to no side effects or toxicity [3]. Several populations in southern African countries utilize *C*. *molle* in traditional medicinal treatments of a number of diseases [11]. This plant’s medicinal properties are related to the plethora of bioactive compounds present within [17]. The results of this study revealed the presence of several phytochemicals with potential medicinal properties within the extracts of *C*. *molle*. Following reflux extraction, the leaves of this species notably produced higher metabolite yields than the stems. Given that phytochemicals vary in polarity, this study utilized solvents of varying polarities to yield a maximum extraction of metabolites [25]. The methanolic extracts produced the greatest yield, with yields from the different solvents increasing with increasing polarity (Table 1). A possible explanation for this may be related to methanol’s ability to enter plant cell walls, resulting in the extraction of both polar and non-polar compounds from within and thereby obtaining a high yield [26]. The yields of *C*. *molle* produced in a study by Simon et al. (2012a) were comparable, with the methanolic extracts having a greater mass than extracts produced by less polar solvents, suggesting that this species contains a wider variety of polar phytochemicals [27].

In the present study, all the extracts (leaves and stems extracted in hexane, chloroform and methanol) produced positive reactions that confirmed the presence of alkaloids, flavonoids, phenolic compounds, polyphenols, terpenoids, tannins, coumarins, saponins, phytosterols, gums, mucilage, carbohydrates, amino acids and proteins (Table 2). The methanolic leaf and stem extracts additionally produced intense positive results for lipids and fixed oils, resulting in these extracts having the highest variety of phytocompounds. This finding was unanticipated as lipidic substances are generally prominent in solvents of lower polarity due to the non-polar and amphipathic nature of the compound [28]. Furthermore, only the methanolic extracts presented positive reactions for saponins via the olive oil test, although the hexane and chloroform extracts did present saponins via the foam test. In addition, the methanol extracts produced intense positive reactions for alkaloids when tested with Wagner’s reagent. These findings may be attributed to the high polarity of methanol which has a higher extraction capacity for saponins and alkaloids [29]. The phytocompounds detected in the present study provide insights on the use of *C*. *molle* in traditional medicine due to the several therapeutic properties that these compounds possess. This species contained several major secondary metabolite classes that exhibit medicinal properties, including alkaloids, phenolic compounds and terpenoids. Alkaloids are nitrogen-containing secondary metabolites [30] which produce anesthetic, analgesic, antihypertensive, antibacterial, anticancer, antidiabetic, antimalarial, antioxidant and anti-inflammatory effects [31]. These compounds offer plants protection from herbivory and microbial colonization [10]. Compounds such as flavonoids, polyphenols, tannins, coumarins and lignins classify as phenolic compounds and are categorized by having one or more aromatic rings with hydroxyl groups [32]. These phytochemicals have antioxidant, antibacterial, anticancer, cardioprotective and anti-inflammatory properties [33]. Furthermore, polyphenols are often involved in the treatment of chronic diseases such as diabetes and cancer [34]. In general, phenolic compounds offer protection to plants from ultraviolet radiation, pathogens and herbivory [35,36], with compounds such as lignin being indigestible to herbivores, thus minimizing plant consumption [37]. Terpenoids are a diverse group of compounds derived from 5-carbon isoprene units and include terpenes, saponins, resins, phytosterols and essential oils, among several other compounds [38]. These phytocompounds additionally possess anti-inflammatory, antimalarial, antibacterial and anticancer activities [39]. With regards to plant defense, terpenoids may deter insects by olfactory mechanisms [40], while sesquiterpenes may produce a bitter taste, rendering plants inedible to certain insects [41]. Furthermore, terpenes reportedly attract pollination agents as well as mites that may aid in the deterrence of herbivores [42].

The curative properties of the phytochemicals present within extracts of *C*. *molle* support the medicinal use of this species, with emphasis on the involvement of this plant in the treatment of venereal diseases, swelling, diarrhea and malaria [11,20]. Studies have further revealed the plant’s activity against T-24 bladder cancer cells [43], as well as its potential antidiabetic role in lowering blood glucose levels and elevating insulin [44]. *Combretum molle* has also been reported to possess cardioprotective properties [45], possibly due to the presence of mollic acid glucoside (triterpene acid saponin) [11,21]. Despite being essential in plants, the presence of common primary metabolites (e.g., carbohydrates, proteins and lipids) in the extracts of *C*. *molle* may also be important in medicinal applications and plant defense. The use of plant-derived carbohydrates, such as gums and mucilage, in the formulation of tablets as binding and thickening agents, as well as in drug delivery, offers a safer alternative to the use of synthetic polymers [46]. Mucilage further plays a role in relieving inflammation of the gastrointestinal tract [46], supporting the traditional use of *C*. *molle* in the treatment of diarrhea and other abdominal disorders. Furthermore, although necessary for enzymatic, structural and functional roles, proteins may serve in plant defense by the formation of enzyme complexes which deter pathogens, insects and herbivores [47]. In addition, the presence of lipidic substances implies the plant’s protective mechanism against water loss and pathogenic organisms [48]. Overall, screening of the *C*. *molle* leaves and stems in this study revealed analogous phytochemical constituents, with the same phytochemicals being detected within each organ. A recent phytochemical investigation of *Combretum erythrophyllum* by Bantho et al. [49] reported a greater variety of phytochemicals within the stembark than the leaf, while a study by Sousa et al. [50] showed that various fractions of ethanolic extracts of the leaves and stembark of *Combretum leprosum* yielded comparable phytochemical constituents. The presence of the same compounds within more than one organ indicates the potential for synergistic use of the leaf and stem of *C*. *molle* in medicinal treatment. 

Remarkably, resins and glycosides were absent in all extracts of *C*. *molle*. Another study on the aqueous and organic extracts of the plant’s leaves reported contrasting findings with positive results for both phytocompounds [51]. In addition, Kulawe et al. [52] reported the presence of glycosides in ethyl-acetate fractions of the *C*. *molle* root, and glycosides were additionally isolated from aqueous-methanolic extracts of the plant’s stembark [53]. Nevertheless, findings from a study by Saidu and Abdullahi [54] were comparable to those of the present study, where aqueous, ethanol and methanol extracts of *C*. *molle* leaves produced negative results for glycosides. With regards to the rest of the phytoconstituents detected in the present study, another investigation on the ethanolic extracts of leaves of *C*. *molle* produced similar findings with the detection of tannins, flavonoids, saponins and phenols; however, that study also reported the absence of alkaloids [55]. The differences in phytochemical composition within the species may be attributed to variations in locality, climate, season and extraction methods [56,57]. Several reports on the phytochemical composition of other *Combretum* species including *C*. *erythrophyllum*, *C*. *leprosum*, *Combretum album* and *Combretum glutinosum* revealed the presence of similar phytoconstituents to the present investigation [49,50,58,59]. 

### 3.2. Characterization of Powdered Samples and FTIR Spectroscopy

Characteristic evaluations of powdered plant material are essential in the quality control of medicinal plants [24,60]. The use of *C*. *molle* in traditional medicine and the need to validate this species in modern medicine has led to the need for pharmacognostic evaluations to prevent adulteration and maintain the high quality of products [24]. FTIR spectroscopy is a non-destructive analytical technique that is essentially used to identify chemical compounds present within a sample by its unique spectral fingerprint [61]. In this study, FTIR spectroscopic analyses were conducted to confirm the presence of phytocompounds within powdered leaf and stem samples of *C*. *molle* by identifying the functional groups present. The FTIR spectra (Figure 1) revealed prominent peaks corresponding to the functional groups of several chemical compounds, including alcohols, phenols, primary amines, alkyl halides (halo compounds), alkanes and alkyl aryl ethers (Table 3). This suggests the presence of phytochemicals, such as alkaloids, phenolic compounds, flavonoids, saponins, terpenes, carbohydrates and lipids [62], which complements the findings from the qualitative phytochemical screening of the plant’s extracts. FTIR spectroscopy revealed that both leaf and stem powdered material contained alcohols and phenolic compounds, as inferred by the presence of hydroxyl groups in the regions of 3200–3570 cm^−1^ and 1310–1410 cm^−1^ [63,64], respectively. Previous studies have proposed that the detection of alcoholic compounds may be a result of the presence of carbohydrates (polysaccharides), polyphenols (lignins), organic acids and terpenoids (monoterpenes) within the plant material [62]. In addition, the hydroxyl groups that correspond to phenols may be attributed to the presence of simple phenolics, flavonoids, coumarins, tannins and lignans [62]. The hydroxyl groups in phenolic compounds are highly important for a compound’s antimicrobial and antioxidant effects [65], thus suggesting the role of phenolic compounds in the medicinal activities in *C*. *molle* [17,55]. The leaf and stem spectra additionally resulted in the detection of primary amines at wavelengths between 1590 and 1650 cm^−1^ (N-H bending) and 1020 and 1090 cm^−1^ (C-N stretching) [63,64]. This correlates with the presence of alkaloids which characteristically contain one or more nitrogen atoms in a heterocyclic ring [66]. Both the leaf and stem were further comprised of alkyl halides, with iodine groups occurring from 500 to 600 cm^−1^ [63,64]. Alkyl halides are known to confer significant antimicrobial activity [67], giving credence to the use of *C*. *molle* as an antibacterial and antifungal agent [68]. Similar findings were obtained for dried aqueous leaf extracts of *Combretum indicum*, where alkyl halides with an iodine group were detected from 580 to 675 cm^−1^ [69]. In the present study, alkyl halides with chlorine groups were additionally detected from 700 to 800 cm^−1^ in the stem [63,64]. The detection of similar functional groups in the leaves and stems complemented the results of the qualitative phytochemical screening, which revealed similar phytochemical constituents within the leaves and stems of *C*. *molle*. Nevertheless, the FTIR spectra of the powdered leaf illustrated a greater variety of functional groups than the stem, and additionally, consisted of alkanes between 2915 and 2935 cm^−1^, and alkyl aryl ethers between 1230 and 1270 cm^−1^ [63,64]. Alkanes form part of the leaf epicuticular wax covering, providing a feasible explanation for the absence of the compound in the stem [70]. A recent report indicated that absorbance between 2800 and 3000 cm^−1^ corresponds to the C-H group of alkanes including lipidic substances [71]. Similar to alkyl halides, alkanes additionally have a significant role in a plant’s antimicrobial activity [68]. Ether bonds occur in phytocompounds such as lipids, terpenoids, flavonoids and carbohydrate derivatives [72]. Alkyl aryl ethers are used in the production of fragrances, cosmetics and pharmaceuticals, justifying the potential use of *C*. *molleole* in medicinal applications [73]. 

A study conducted by Ntshanka et al. [17] on leaf extracts of *C*. *molleole* resulted in dissimilar results, potentially due to the use of extracts rather than powdered material [17]. The study revealed functional groups corresponding to alkenes, phenolics, carbonyl and methylene groups at 1594, 1012, 1705, and 2915 cm^−1^, respectively. Similar results to the present study were, however, found at 3228 cm^−1^ where alcoholic hydroxyl groups were detected. Overall, these findings confirmed the presence of phenols, flavonoids and tannins within the plant’s extracts. Additionally, there are many variations and similarities in FTIR spectra among species of *Combretum*. An analysis on *C*. *indicum* dried aqueous leaf extracts revealed aromatic compounds between 3029 and 3073 cm^−1^, nitric oxide compounds from 1492 to 1586 cm^−1^ and ethers at 1050 cm^−1^ [69]. FTIR spectroscopy conducted on hydroalcoholic leaf extracts of *Combretum micranthum* revealed similar functional groups as the present study, including hydroxyl compounds and ethers [74]. Another investigation on *C*. *erythrophyllum* leaf aqueous extracts revealed comparable findings with hydroxyl groups detected at 3260 cm^−1^ and alkanes at 2932 cm^−1^ [75]. 

### 3.3. Elemental Composition

Mineral elements are required by plants for the maintenance of physiological and growth processes [76]. Determining the elemental composition of medicinal plants is key to understanding their metabolism and physiology. Upon human consumption, some of these elements are involved in the facilitation of metabolic processes, while others assume a nutritive and/or medicinal role [77]. Several metal elements serve as cofactors or enzymatic activators in the catalysis and modulation of the metabolism of natural products and are, therefore, essential in medicinal plants [78]. However, assimilation of heavy metals, toxic compounds and an excess of certain mineral elements may result in several disorders [79]. It is, therefore, essential to determine the elemental composition of these plants prior to consumption. Hence, the present study conducted EDX microanalyses to identify the elements present within the powdered leaf and stem samples of *C*. *molle* at a microscopic level. Each element has a characteristic atomic structure that results in specific peaks on its spectra of electromagnetic emissions upon excitation by an X-ray source, thereby forming the EDX spectra that illustrates the elemental composition of the plant [80]. In this study, EDX microanalyses revealed that essential non-mineral macro-elements (carbon and oxygen) constituted the majority of the leaf and stem material of *C. molle*, as anticipated. The plant additionally comprised several essential mineral elements (macro- and trace elements). A wider variety of elements were present within the leaves, which contained calcium, chlorine, magnesium, potassium, sodium, manganese and rubidium, while the stem comprised calcium, magnesium and manganese (Figure 2, Table 4). A study by Mtunzi et al. (2012) determined the concentration of mineral elements within the leaves of *C*. *molle* and found that calcium and magnesium were present in large quantities [81].

According to Aliyu et al. [82], calcium and potassium play a role in the prevention and control of diseases. The presence of calcium may be attributed to the occurrence of calcium crystals which are common within medicinal plant species [83], although, at present, there are no reports on the presence of these crystals within *C*. *molle.* Oxalate crystals are ideal for the identification of adulterants due to variations in their morphological structure [83]. Calcium crystals reportedly occur in several species of *Combretum* including *C*. *erythrophyllum*, *Combretum collinum*, *C*. *glutinosum*, *Combretum paniculatum* and *Combretum zenkeri* [35,84]. Chlorine serves several metabolic and physiological purposes in plant leaves by its involvement in stomatal regulation and in the Hill reaction of photosystem II [85], which explains the absence of chlorine within the stem. Magnesium and potassium are essential components in the maintenance of a plant’s physiological and metabolic processes, with metabolite yield being related to its content [86,87]. Although needed with potassium to maintain enzymatic reactions required for plant growth and development, sodium ions in excess quantities may hinder plant metabolism [88]. Similarly, the presence of heavy metals within plants may result in fluctuations in metabolite yield [89]. Therefore, it is imperative to ensure that medicinal plants are grown and harvested in soil with sufficient minerals that result in optimum metabolism, as well as in vicinities devoid of heavy metal contamination [90]. Furthermore, manganese has been reported to modulate the role of bioactive compounds in medicinal plants as it is involved in the initial stage of phenolic compound biosynthesis, as well as in the synthesis of alkaloids and terpenoids [78]. Variations in the elemental composition fluctuates with species; however, environmental conditions (e.g., edaphic factors, soil type, rainfall, water content and temperature) and pollutants from agricultural and industrial practices influence the elemental composition of plants [91]. For instance, the presence of rubidium within the leaves of *C*. *molle* was unanticipated due to the element being characterized as “ultra-trace” [92]. However, according to Kosla et al. [93], the occurrence of rubidium may be attributed to a low soil pH. At high concentrations, potassium generally inhibits a plant’s rubidium uptake; however, the minute quantities of potassium present within the leaves in the present study may explain the assimilation of rubidium [93]. A few of the mineral elements found in the present study correspond to those found in other *Combretum* species. A recent analysis on the elemental composition of powdered material of *C*. *erythrophyllum* by Bantho et al. [49] revealed a similar elemental composition with small quantities of chlorine, potassium, calcium and magnesium in the leaves, while the stembark contained chlorine, potassium and calcium. Another study which investigated the mineral elements found within the leaves of *C*. *zenkeri* detected calcium, magnesium, manganese, potassium and sodium [94], corresponding to some of the elements found in *C*. *molle*.

### 3.4. Fluorescence Microanalyses

Fluorescence microanalyses on powdered medicinal plants are essential to validate and authenticate the identity, quality and purity of a species by identifying specific structural characteristics and phytocompounds under fluorescence [24,59]. The fluorescence microanalyses in the present study revealed that upon the addition of various reagents, the leaf and stem material of *C*. *molle* underwent distinct colour changes that fluoresced under ultraviolet light (Figure 3 and Figure 4, Table 5). Although some plant constituents and phytochemicals fluoresce under visible light, others fluoresce only under ultraviolet light [95]. Therefore, various reagents are added to the material to convert substances that do not fluoresce naturally into fluorescent derivatives or decomposition products, resulting in the distinct colour changes as observed in this study [24,59]. Similar findings were noted in *C*. *erythrophyllum* and *Combretum albidum* by Bantho et al. [49] and Zalke et al. [96], respectively, where fluorescent colour changes were observed in the plant material when viewed under ultraviolet light when treated with various reagents. In addition to the colour changes noted in the present study, characteristic peltate glandular scales, non-glandular trichomes and cellular segments of the *C*. *molle* leaf and stem were observed. These observations are essential for the establishment of standard characteristics that may be used to identify *C*. *molle* in its powdered form. Furthermore, such analyses may avert the misidentification of species and also enable adulterants to be detected, thereby preserving the quality and efficacy of medicinal plants while also protecting consumers of these products [59].

## 4. Materials and Methods

### 4.1. Plant Material Collection and Extract Preparation

Leaves and stems of *C*. *molle* were collected from a single tree located in a roadside garden on Pitlochry Road (29°49.0985’ S, 30°56.1057′ E), Westville North, Durban, KwaZulu-Natal, South Africa. A voucher specimen was deposited at the Bews Herbarium in the School of Life Sciences, University of KwaZulu-Natal, Pietermaritzburg Campus, with accession no. NU0092543, collected by M. Parusnath (collector no. 1). The collected leaves and stems were dried in an oven (EcoTherm, Labotec, Durban, South Africa) for three weeks at 30 °C. The dried material was ground separately into fine powders using a blender (Russell Hobbs, RHB048, Failsworth, Manchester, UK). Subsequently, 10 g each of the leaf and stem powdered material were placed into separate 250 mL round-bottom flasks to which 100 mL of solvent was added. Using a reflux apparatus, the solutions were boiled thrice for three hours, each with an intervening filtration step using a Whatman^®^ No. 1 filter paper. Three different solvents were used; therefore, the reflux process was repeated for each solvent in order of increasing polarity, i.e., hexane, chloroform, and then methanol (Merck, Darmstadt, Germany). The filtrates were allowed to evaporate under ventilated conditions at room temperature in the dark. Upon complete evaporation, the filtrates were stored in hermetic glass bottles. The percentage yields for each of the leaf and stem hexane, chloroform and methanol extracts were determined using the following formula:

(1) $$\mathrm{Extract}\;\mathrm{yield}\;(\%)=\frac{\mathrm{mass}\;\mathrm{of}\;\mathrm{dried}\;\mathrm{extract}\;(g)}{\mathrm{mass}\;\mathrm{of}\;\mathrm{powdered}\;\mathrm{material}\;(g)}\times \;100;$$

where mass of dried extract is the difference in extract weight before and after evaporation, and mass of powdered material is the mass of leaves and stems that were used to produce the extracts (10 g).

### 4.2. Qualitative Phytochemical Screening

For each of the leaf and stem hexane, chloroform and methanol extracts, the phytochemical screening of alkaloids, flavonoids, phenolic compounds, polyphenols, terpenoids, tannins, coumarins, saponins, phytosterols, gums and mucilage, resins, glycosides, carbohydrates, amino acids and proteins, and lipids and fixed oils were performed according to Harborne [97], Trease and Evans [98] and Sofowora [99], with minor adjustments in some cases according to Bantho et al. [49]. The analyses were conducted in triplicate, along with the appropriate standard control procedures.

### 4.3. Preparation of Powdered Samples

The fresh leaves and stems were oven dried (EcoTherm, Labotec, South Africa) for three weeks at 30 °C. Thereafter, the dried material was ground separately into fine powders using a blender (Russell Hobbs RHB048, Failsworth, Manchester, UK). The powdered material was stored in hermetic glass bottles for use in the subsequent FTIR, EDX and fluorescence microanalyses.

### 4.4. Fourier Transform Infrared (FTIR) Spectroscopy

The powdered leaf and stem samples of *C*. *molle* were subjected to Fourier transform infrared spectroscopy on a Perkin Elmer Spectrum 100 FT-IR Spectrometer (Shelton, Connecticut, USA), software version 6.1. The powdered samples were scanned from a wavelength of 400 to 4000 cm^−1^, with a spectral resolution of 4 cm^−1^ and at a temperature of 25–27 °C. The detected characteristic peak frequencies were compared with reference literature to determine the functional groups of the phytochemicals present within each sample [63,64].

### 4.5. Energy-Dispersive X-ray (EDX) Microanalyses

To determine the plant’s elemental composition, the powdered leaf and stem samples were subjected to EDX microanalyses. The powdered material was secured on aluminium stubs using carbon conductive tape and sputter-coated with gold using a Quorum Q150 GB Gold Coater (Laughton, East Sussex, UK). The samples were analyzed using an EDX detector on a Zeiss Ultra Plus FEG SEM (Smart SEM) (Oberkochen, Germany) at 5 kV, equipped with Aztec Analysis Software (Oxford Instruments, High Wycombe Buckinghamshire, UK).

### 4.6. Fluorescence Microanalyses

The fluorescence activities of the powdered leaf and stem samples of *C*. *molle* were investigated by analyzing combinations of the powdered material with various reagents under ultraviolet light. These reagents included distilled water, hexane, chloroform, methanol, ethanol, acetic acid, sodium hydroxide, sulphuric acid, hydrochloric acid and acetone. Powdered material with no reagent served as the control. Approximately 0.1 g of each powdered sample was placed separately onto clean glass slides. Thereafter, two drops of the respective reagent were added and the sample was mixed by gently tilting the slide. The samples were left to stand in the dark for three min, allowing the reagents to thoroughly infiltrate the powdered material. The slides were viewed and photographed using a Nikon Eclipse 80i Compound Microscope (Japan) with a Nikon DS Fi1 camera equipped with NIS Elements D Imaging Software Version 4.6, under brightfield and ultraviolet-2A (UV-2A) light at wavelengths of 330–380 nm.

## 5. Conclusions

Phytochemical screening revealed the medicinal properties of *C*. *molle* leaf and stem extracts. The methanol extracts produced the greatest variety of phytochemicals including alkaloids, flavonoids, phenolic compounds, polyphenols, terpenoids, tannins, coumarins, saponins, phytosterols, gums, mucilage, carbohydrates, amino acids and proteins, lipids and fixed oils. The powdered material of *C*. *molle* presented several functional groups related to these phytocompounds and additionally contained essential elements that are required for the optimum functioning of medicinal plants. These findings give credence to the use of *C*. *molle* in traditional medicine and highlight the potential medicinal use of this species and its biological activities. Further quantitative analyses and a full nutritive and proximate analysis on this species should be performed. Lastly, safety and toxicity studies are required to establish the safe amounts of the plant for consumption and treatment.

## Figures and Tables

**Table 1:** Percentage yield of hexane, chloroform and methanol leaf and stem extracts of *Combretum molle*.

Solvent	Extract Yield (%)	
	Leaf	Stem
Hexane	7.00	4.30
Chloroform	7.10	6.50
Methanol	12.90	11.30

**Table 2:** Preliminary qualitative phytochemical screening of the hexane, chloroform and methanol leaf and stem extracts of *Combretum molle*.

Phytocompound	Test	Extract					
		Leaf			Stem		
		H	C	M	H	C	M
Alkaloids	Wagner’s test	+	+	++	+	+	++
	Mayer’s test	+	+	+	+	+	+
Flavonoids	Lead acetate	++	++	++	++	++	++
	Alkaline reagent test	+	+	+	+	+	+
	Acid hydrolysis test	+	+	+	+	+	+
Phenolic compounds	Ferric chloride test	+	+	+	+	+	+
	Lead acetate test	+	+	+	+	+	+
Polyphenols	Ferric chloride test	+	+	+	+	+	+
Terpenoids	Salkowski’s test	+	+	+	+	+	+
	Liebermann–Burchard test	+	+	+	+	+	+
Tannins	Ferric chloride test	+	+	+	+	+	+
	Lead acetate test	+	+	+	+	+	+
	Gelatin test	+	+	+	+	+	+
Coumarin	Sodium hydroxide test	+	+	+	+	+	+
Saponin	Foam test	+	+	+	+	+	+
	Olive oil test	–	–	+	–	–	+
Phytosterols	Salkowski’s test	+	+	+	+	+	+
	Liebermann–Burchard test	+	+	+	+	+	+
Gums and mucilage	Precipitation test	+	–	–	+	–	–
	Ruthenium red test	+	+	+	+	+	+
Resins	Acetone test	–	–	–	–	–	–
Glycosides	Keller–Killani test	–	–	–	–	–	–
Carbohydrates	Molisch’s test	+	+	+	+	+	+
	Fehling’s test	+	+	+	+	+	+
Amino acids and proteins	Ninhydrin test	+	+	+	+	+	+
	Biuret test	+	+	+	+	+	+
Lipids and fixed oils	Spot test	–	–	++	–	–	++

Note: (++) = intense positive reaction; (+) = positive reaction (–) = negative reaction; H = hexane; C = chloroform; and M = methanol.

**Table 3:** FTIR spectral classification of powdered leaves and stems of *Combretum molle*.

Sample	Peak Absorption Frequency (cm^−1^)	Intensity	Functional Group	Compound Class	Type of Vibration
Leaf	3283.18	strong, broad	O-H	alcohol	stretching
	2917.81	medium	C-H	alkane	stretching
	1617.72	medium	N-H	primary amine	bending
	1318.83	medium	O-H	phenol	bending
	1233.97	strong	C-O	alkyl aryl ether	stretching
	1032.32	medium	C-N	primary amine	stretching
	521.38	strong	C-I	alkyl halide (halo compound)	stretching
Stem	3318.91	strong, broad	O-H	alcohol	stretching
	1619.25	medium	N-H	primary amine	bending
	1317.13	medium	O-H	phenol	bending
	1032.68	medium	C-N	primary amine	stretching
	780.86	strong	C-Cl	alkyl halide (halo compound)	stretching
	516.39	strong	C-I	alkyl halide (halo compound)	stretching

**Table 4:** Elemental composition (%) of powdered leaf and stem samples of *Combretum molle*.

Element	Composition (%)	
	Leaf	Stem
C	68.44	54.92
O	26.72	42.86
Na	0.13	–
Mg	0.93	0.43
Cl	0.96	–
K	0.71	–
Ca	1.87	1.70
Mn	0.12	0.09
Rb	0.10	–

Note: C = carbon; O = oxygen; Na = sodium; Mg = magnesium; Cl = chlorine; K = potassium; Ca = calcium; Mn = manganese; Rb = rubidium; and (–) = absent.

**Table 5:** Fluorescence analyses of powdered leaf and stem samples of *Combretum molle*.

Treatment	Sample			
	Leaf		Stem	
	Brightfield	UV Light	Brightfield	UV Light
Powder only (control)	Green	Multicoloured fluorescence	Brown	Green and light blue fluorescence
Powder and distilled water	Green	Green with blue and red fluorescence	Brown	Orange with blue and purple fluorescence
Powder and hexane	Green	Green with pink and blue fluorescence	Brown	Green with blue and gold fluorescence
Powder and chloroform	Green	Blue, pink, green and purple fluorescence	Brown	Multicoloured fluorescence
Powder and methanol	Green	Green with blue and orange fluorescence	Brown	Green, blue and purple fluorescence
Powder and ethanol	Green	Green with multicoloured fluorescence	Brown	Multicoloured fluorescence
Powder and acetic acid	Green	Pink, purple and orange fluorescence	Brown	Brown with blue and purple fluorescence
Powder and sodium hydroxide	Green	Blue and red fluorescence	Brown	Blue and turquoise fluorescence
Powder and sulphuric acid	Green	Blue, purple and pink fluorescence	Brown	Blue fluorescence
Powder and hydrochloric acid	Green	Green with yellow and blue fluorescence	Brown	Green with yellow and blue fluorescence
Powder and acetone	Green	Gold with blue and orange fluorescence	Brown	Gold with blue and orange fluorescence

## Data Availability

All data are presented in the article.

## References

[1] Wink M. (2015). Modes of action of herbal medicines and plant secondary metabolites. Medicines.

[2] Agidew M.G. (2022). Phytochemical analysis of some selected traditional medicinal plants in Ethiopia. Bull. Natl. Res. Cent..

[3] Farooq S., Ngaini Z. (2021). Natural and synthetic drugs as potential treatment for coronavirus disease 2019 (COVID-2019). Chem. Afr..

[4] Kengne I.C., Feugap L.D.T., Njouendou A.J., Ngnokam C.D.J., Djamalladine M.D., Ngnokam D., Voutquenne-Nazabadioko L., Tamokou J.-D.-D. (2021). Antibacterial, antifungal and antioxidant activities of whole plant chemical constituents of *Rumex abyssinicus*. BMC Complement. Med. Ther..

[5] Abdullahi A.A. (2011). Trends and challenges of traditional medicine in Africa. Afr. J. Tradit. Complement. Altern. Med..

[6] Moeti M. African Traditional Medicine Day 2022. https://www.afro.who.int/regional-director/speeches-messages/african-traditional-medicine-day-2022.

[7] Jalali A., Dabaghian F., Akbrialiabad H., Foroughinia F., Zarshenas M.M. (2021). A pharmacology-based comprehensive review on medicinal plants and phytoactive constituents possibly effective in the management of COVID-19. Phytother. Res..

[8] Les F., Cásedas G., López V. (2021). Bioactivity of medicinal plants and extracts. Biology.

[9] Joshi R. (2018). Role of natural products against microorganisms. Am. J. Clin. Microbiol. Antimicrob..

[10] Muravnik L. (2021). The Structural Peculiarities of the Leaf Glandular Trichomes: A Review. Plant Cell and Tissue Differentiation and Secondary Metabolites: Fundamentals and Applications.

[11] Rademan S., Lall N. (2020). Combretum molle, in Underexplored Medicinal Plants from Sub-Saharan Africa: Plants with Therapeutic Potential for Human Health.

[12] Van Wyk B. (2013). Field Guide to Trees of Southern Africa.

[13] Burkill H.M. (1985). The Useful Plants of West Tropical Africa.

[14] Andefiki U., Ileigo I.H., Isah A. (2017). In Vivo Activity of Fractions of *Combretum molle* R. and Haematological Profile of *Trypanosoma Brucei Brucei* Infected Mice. Am. J. Pharm. Pharmacol..

[15] Jordaan M., Van Wyk A.E.B., Maurin O. (2011). A conspectus of *Combretum* (Combretaceae) in southern Africa, with taxonomic and nomenclatural notes on species and sections. Bothalia.

[16] Bein E., Jaber A., Birnie A., Tengnäs B. (1996). Useful Trees and Shrubs in Eritrea.

[17] Ntshanka N.M., Ejidike I.P., Mthunzi F.M., Moloto M.J., Mubiayi K.P. (2020). Investigation into the phytochemical profile, antioxidant and antibacterial potentials of *Combretum molle* and *Acacia mearnsii* leaf parts. Biomed. Pharmacol. J..

[18] Pegel K.H., Rogers C.B. (1985). The characterisation of mollic acid 3β-D-xyloside and its genuine aglycone mollic acid, two novel 1α-hydroxycycloartenoids from *Combretum molle*. J. Chem. Soc. Perkin Trans..

[19] Ponou B.K., Barboni L., Teponno R.B., Mbiantcha M., Nguelefack T.B., Park H.-J., Lee K.-T., Tapondjou L.A. (2008). Polyhydroxyoleanane-type triterpenoids from *Combretum molle* and their anti-inflammatory activity. Phytochem. Lett..

[20] Asres K., Bucar F., Knauder E., Yardley V., Kendrick H., Croft S. (2001). In vitro antiprotozoal activity of extract and compounds from the stem bark of *Combretum molle*. Phytother. Res..

[21] Ojewole J.A. (2008). Cardiovascular effects of mollic acid glucoside, a 1α-hydroxycycloartenoid saponin extractive from *Combretum molle* R Br ex G Don (Combretaceae) leaf. Cardiovasc. J. Afr..

[22] Asres K., Bucar F. (2005). Anti-HIV activity against immunodeficiency virus type 1 (HIV-I) and type II (HIV-II) of compounds isolated from the stem bark of *Combretum molle*. Ethiop. Med. J..

[23] Afreed Muhammed N., Ranjith D., Vinayakraj M., Rahman M., Sivan V., Sanis J. (2018). Physical characteristics, extractive yield and qualitative phytochemical analysis of *Flueggea leucopyrus* Willd leaves. J. Med. Plants.

[24] Chanda S. (2014). Importance of pharmacognostic study of medicinal plants: An overview. J. Pharmacogn. Phytochem..

[25] Zaman M.K., Azzeme A.M., Ramli S.N., Shaharuddin N.A., Ahmad S., Abdullah S.N.A. (2020). Solvent extraction and its effect on phytoch[1]emical yield and antioxidant capacity of woody medicinal plant, *Polyalthia bullata*. BioResources.

[26] Hasmila I., Natsir H., Soekamto N. (2019). Phytochemical analysis and antioxidant activity of soursop leaf extract (*Annona muricata* Linn.). J. Phys. Conf. Ser..

[27] Simon M., Ajanusi O., Abubakar M., Idris A., Suleiman M. (2012). The anthelmintic effect of aqueous methanol extract of *Combretum molle* (R. Br. x. G. Don) (Combretaceae) in lambs experimentally infected with *Haemonchus contortus*. Vet. Parasitol..

[28] Loneman D.M., Peddicord L., Al-Rashid A., Nikolau B.J., Lauter N., Yandeau-Nelson M.D. (2017). A robust and efficient method for the extraction of plant extracellular surface lipids as applied to the analysis of silks and seedling leaves of maize. PLoS ONE.

[29] Yusnawan E. (2013). The effectiveness of polar and non polar fractions of *Ageratum conyzoides* l. to control peanut rust disease and phytochemical screenings of secondary metabolites. J. Trop. Plant Pests Dis..

[30] Lichman B.R. (2021). The scaffold-forming steps of plant alkaloid biosynthesis. Nat. Prod. Rep..

[31] Gutiérrez-Grijalva E.P., López-Martínez L.X., Contreras-Angulo L.A., Elizalde-Romero C.A., Heredia J.B. (2020). Plant Alkaloids: Structures and Bioactive Properties. Plant-Derived Bioactives.

[32] Dai J., Mumper R.J. (2010). Plant phenolics: Extraction, analysis and their antioxidant and anticancer properties. Molecules.

[33] Tungmunnithum D., Thongboonyou A., Pholboon A., Yangsabai A. (2018). Flavonoids and other phenolic compounds from medicinal plants for pharmaceutical and medical aspects: An overview. Medicines.

[34] Pandey K.B., Rizvi S.I. (2009). Plant polyphenols as dietary antioxidants in human health and disease. Oxidative Med. Cell. Longev..

[35] Bantho S., Naidoo Y., Dewir Y. (2020). The secretory scales of *Combretum erythrophyllum* (Combretaceae): Micromorphology, ultrastructure and histochemistry. S. Afr. J. Bot..

[36] Albuquerque B.R., Heleno S.A., Oliveira M.B.P., Barros L., Ferreira I.C. (2021). Phenolic compounds: Current industrial applications, limitations and future challenges. Food Funct..

[37] Chahil G.S., Gill H.K., Goyal G. (2018). Food Chains and Webs: Interaction with Ecosystem. Advances in Crop Environment Interaction.

[38] Zeng T., Chen Y., Jian Y., Zhang F., Wu R. (2022). Chemotaxonomic investigation of plant terpenoids with an established database (TeroMOL). New Phytol..

[39] Jahangeer M., Fatima R., Ashiq M., Basharat A., Qamar S.A., Bilal M., Iqbal H. (2021). Therapeutic and biomedical potentialities of terpenoids—A Review. J. Pure Appl. Microbiol..

[40] Ascensão L., Pais M. (1998). The leaf capitate trichomes of *Leonotis leonurus*: Histochemistry, ultrastructure and secretion. Ann. Bot..

[41] Heinrich G., Pfeifhofer H., Stabentheiner E., Sawidis T. (2002). Glandular hairs of *Sigesbeckia jorullensis* Kunth (Asteraceae): Morphology, histochemistry and composition of essential oil. Ann. Bot..

[42] Zwenger S., Basu C. (2008). Plant terpenoids: Applications and future potentials. Biotechnol. Mol. Biol. Rev..

[43] Fyhrquist P., Mwasumbi L., Vuorela P., Vuorela H., Hiltunen R., Murphy C., Adlercreutz H. (2006). Preliminary antiproliferative effects of some species of *Terminalia*, *Combretum* and *Pteleopsis* collected in Tanzania on some human cancer cell lines. Fitoterapia.

[44] Hamza R.Z., Al-Baqami N.M., Khojah E., Mansour A.M., E. Al-Motaani S., A. Al-Salmi F., El-Megharbel S.M. (2021). Possible antioxidant and antidiabetic effects of *Combretum molle* extract in a diabetes mellitus experimental model in male rats. Nat. Prod. Commun..

[45] Miaffo D., Wansi S.L., Ntchapda F., Kamanyi A. (2020). Chronic oral safety study of the aqueous extract of *Combretum molle* twigs on biochemical, haematological and antioxidant parameters of Wistar rats. BMC Complement. Med. Ther..

[46] Amiri M.S., Mohammadzadeh V., Yazdi M.E.T., Barani M., Rahdar A., Kyzas G.Z. (2021). Plant-based gums and mucilages applications in pharmacology and nanomedicine: A review. Molecules.

[47] Schilmiller A.L., Last R.L., Pichersky E. (2008). Harnessing plant trichome biochemistry for the production of useful compounds. Plant J..

[48] Yeats T.H., Rose J.K. (2013). The formation and function of plant cuticles. Plant Physiol..

[49] Bantho S., Naidoo Y., Dewir Y.H., Bantho A., Murthy H.N. (2022). Chemical Composition of *Combretum erythrophyllum* Leaf and Stem Bark Extracts. Horticulturae.

[50] Sousa H.G., Uchôa V.T., Cavalcanti S.M.G., de Almeida P.M., Chaves M.H., Lima Neto J.D.S., Nunes P.H.M., da Costa Júnior J.S., Rai M., Do Carmo I.S. (2021). Phytochemical screening, phenolic and flavonoid contents, antioxidant and cytogenotoxicity activities of *Combretum leprosum* Mart. (Combretaceae). J. Toxicol. Environ. Health Part A.

[51] Oloya B., Namukobe J., Ssengooba W., Afayoa M., Byamukama R. (2022). Phytochemical screening, antimycobacterial activity and acute toxicity of crude extracts of selected medicinal plant species used locally in the treatment of tuberculosis in Uganda. Trop. Med. Health.

[52] Kulawe D., Muhammad I., Yuguda U.A. (2020). Antibacterial effect of root fractions of *Combretum molle* (R. Br. Ex. G. Don) against selected pathogens. Bima J. Sci. Technol..

[53] Simon M., Nafarnda W., Obeta S. (2012). Iridoid Glycosides Isolated from *Combretum molle* Stem Bark Aqueous Methanol Extract. Glob. Vet..

[54] Saidu T., Abdullahi M. (2011). Phytochemical determinations and antibacterial activities of the leaf extracts of *Combretum molle* and *Gossypium arboretum*. Bayero J. Pure Appl. Sci..

[55] Koevi K.-K.A., Millogo V., Fokou J.B.H., Sarr A., Ouedraogo G.A., Bassene E. (2015). Phytochemical analysis and antioxidant activities of *Combretum molle* and *Pericopsis laxiflora*. Int. J. Biol. Chem. Sci..

[56] Dutta S., Ray S. (2020). Comparative assessment of total phenolic content and *in vitro* antioxidant activities of bark and leaf methanolic extracts of *Manilkara hexandra* (Roxb.) Dubard. J. King Saud Univ. -Sci..

[57] Naidoo C.M., Naidoo Y., Dewir Y.H., Singh M., Daniels A.N., El-Ramady H. (2022). In Vitro investigation of the antioxidant and cytotoxic potential of *Tabernaemontana ventricosa* hochst. Ex A. DC. leaf, stem, and latex extracts. Horticulturae.

[58] Burman S., Bhattacharya K., Mukherjee D., Chandra G. (2018). Antibacterial efficacy of leaf extracts of *Combretum album* Pers. against some pathogenic bacteria. BMC Complement. Altern. Med..

[59] Sall C., Ndoye S.F., Dioum M.D., Seck I., Gueye R.S., Faye B., Thiam C.O., Seck M., Gueye P.M., Fall D. (2017). Phytochemical Screening, Evaluation of Antioxidant and Anti-sickling Activities of Two Polar Extracts of *Combretum glutinosum* Leaves. Perr. ex DC. Br. J. Appl. Sci. Technol..

[60] Zhao Z., Liang Z., Ping G. (2011). Macroscopic identification of Chinese medicinal materials: Traditional experiences and modern understanding. J. Ethnopharmacol..

[61] Chen Y., Zou C., Mastalerz M., Hu S., Gasaway C., Tao X. (2015). Applications of micro-fourier transform infrared spectroscopy (FTIR) in the geological sciences—A review. Int. J. Mol. Sci..

[62] Hussein R.A., El-Anssary A.A. (2019). Plants Secondary Metabolites: The Key Drivers of the Pharmacological Actions of Medicinal Plants. Herbal Medicine.

[63] Coates J., Meyers R.A. (2000). Interpretation of infrared spectra, a practical approach. Encyclopedia of Analytical Chemistry.

[64] Stuart B.H. (2004). Infrared Spectroscopy: Fundamentals and Applications.

[65] Bhuyan D.J., Basu A. (2017). Phenolic compounds potential health benefits and toxicity. Utilisation of Bioactive Compounds from Agricultural and Food Waste.

[66] Ziegler J., Facchini P.J. (2008). Alkaloid biosynthesis: Metabolism and trafficking. Annu. Rev. Plant Biol..

[67] Vanitha A., Kalimuthu K., Chinnadurai V., Nisha K.J. (2019). Phytochemical screening, FTIR and GC-MS analysis of aqueous extract of *Caralluma bicolor*–An endangered plant. Asian J. Pharm. Sci..

[68] Asres K., Mazumder A., Bucar F. (2006). Antibacterial and antifungal activities of extracts of *Combretum molle*. Ethiop. Med. J..

[69] Neriyana P.S., Alva V.D. (2020). A green approach: Evaluation of *Combretum indicum* (CI) leaf extract as an eco-friendly corrosion inhibitor for mild steel in 1M HCl. Chem. Afr..

[70] Bush R., McInerney F. (2010). Variation in n-Alkane Distributions of Modern Plants: Questioning Applications of n-Alkanes in Chemotaxonomy and Paleoecology.

[71] Hayat J., Akodad M., Moumen A., Baghour M., Skalli A., Ezrari S., Belmalha S. (2020). Phytochemical screening, polyphenols, flavonoids and tannin content, antioxidant activities and FTIR characterization of *Marrubium vulgare* L. from 2 different localities of Northeast of Morocco. Heliyon.

[72] de María P.D., van Gemert R.W., Straathof A.J., Hanefeld U. (2010). Biosynthesis of ethers: Unusual or common natural events?. Nat. Prod. Rep..

[73] Fuhrmann E., Talbiersky J. (2005). Synthesis of alkyl aryl ethers by catalytic Williamson ether synthesis with weak alkylation agents. Org. Process Res. Dev..

[74] Kpemissi M., Eklu-Gadegbeku K., Veerapur V.P., Potârniche A.-V., Adi K., Vijayakumar S., Banakar S.M., Thimmaiah N., Metowogo K., Aklikokou K. (2019). Antioxidant and nephroprotection activities of *Combretum micranthum*: A phytochemical, *in*-*vitro* and *ex*-*vivo* studies. Heliyon.

[75] Fanoro O.T., Parani S., Maluleke R., Lebepe T.C., Varghese R.J., Mgedle N., Mavumengwana V., Oluwafemi O.S. (2021). Biosynthesis of Smaller-Sized Platinum Nanoparticles Using the Leaf Extract of *Combretum erythrophyllum* and its Antibacterial Activities. Antibiotics.

[76] Ma J.F., Tsay Y.-F. (2021). Transport systems of mineral elements in plants: Transporters, regulation and utilization. Plant Cell Physiol..

[77] Kumar M., Puri S., Pundir A., Bangar S.P., Changan S., Choudhary P., Parameswari E., Alhariri A., Samota M.K., Damale R.D. (2021). Evaluation of nutritional, phytochemical, and mineral composition of selected medicinal plants for therapeutic uses from cold desert of Western Himalaya. Plants.

[78] Lovkova M.Y., Buzuk G., Sokolova S., Kliment’eva N. (2001). Chemical features of medicinal plants. Appl. Biochem. Microbiol..

[79] Kohzadi S., Shahmoradi B., Ghaderi E., Loqmani H., Maleki A. (2019). Concentration, source, and potential human health risk of heavy metals in the commonly consumed medicinal plants. Biol. Trace Elem. Res..

[80] Scimeca M., Bischetti S., Lamsira H.K., Bonfiglio R., Bonanno E. (2018). Energy Dispersive X-ray (EDX) microanalysis: A powerful tool in biomedical research and diagnosis. Eur. J. Histochem..

[81] Mtunzi F., Singo T., Pholosi A., Mzinyane N., Modise J., Sipamla A. (2012). Investigation of the nutritive value and mineral elements of *Combretum molle* leaves. Pak. J. Nutr..

[82] Aliyu A., Musa A., Oshanimi J., Ibrahim H., Oyewale A. (2008). Phytochemical analyses and mineral elements composition of some medicinal plants of Northern Nigeria. Niger. J. Pharm. Sci..

[83] Anitha R., Sandhiya T. (2014). Occurrence of calcium oxalate crystals in the leaves of medicinal plants. Int. J. Pharmacogn..

[84] Ekeke C., Agbagwa I.O. (2014). Ergastic substances (calcium oxalate crystals) in the leaf of *Combretum* Loefl. (Combretaceae) species in Nigeria. Am. J. Plant Sci..

[85] Chen W., He Z.L., Yang X.E., Mishra S., Stoffella P.J. (2010). Chlorine nutrition of higher plants: Progress and perspectives. J. Plant Nutr..

[86] Amtmann A., Rubio F. (2012). Potassium in Plants.

[87] Ishfaq M., Wang Y., Yan M., Wang Z., Wu L., Li C., Li X. (2022). Physiological Essence of Magnesium in Plants and Its Widespread Deficiency in the Farming System of China. Front. Plant Sci..

[88] Wakeel A. (2013). Potassium–sodium interactions in soil and plant under saline-sodic conditions. J. Plant Nutr. Soil Sci..

[89] Ozyigit I.I., Yalcin B., Turan S., Saracoglu I.A., Karadeniz S., Yalcin I.E., Demir G. (2018). Investigation of heavy metal level and mineral nutrient status in widely used medicinal plants’ leaves in Turkey: Insights into health implications. Biol. Trace Elem. Res..

[90] Nkuba L.L., Mohammed N.K. (2017). Heavy metals and essential elements in selected medicinal plants commonly used for medicine in Tanzania. Chem. Sci. Int. J..

[91] Asuk A.A., Agiang M.A., Dasofunjo K., Willie A.J. (2015). The biomedical significance of the phytochemical, proximate and mineral compositions of the leaf, stem bark and root of *Jatropha curcas*. Asian Pac. J. Trop. Biomed..

[92] Anke M., Angelow L., Müller R., Anke S. (2005). Recent progress in exploring the essentiality of the ultratrace element rubidium to the nutrition of animals and man. Biomed. Res. Trace Elem..

[93] Kosla T., Skibniewska E., Debski B., Urbanska-Slomka G. (2002). Rubidium in the trophic chain soil-plants-animals. Trace Elem. Electrolytes.

[94] Ujowundu C., Okafor O., Agha N., Nwaogu L., Igwe K., Igwe C. (2010). Phytochemical and chemical composition of *Combretum zenkeri* leaves. J. Med. Plants Res..

[95] Pandavadra M., Chanda S. (2014). Development of quality control parameters for the standardization of *Limonia acidissima* L. leaf and stem. Asian Pac. J. Trop. Med..

[96] Zalke A.S., Duraiswamy B., Gandagule U.B. (2013). Pharmacognostical studies of leaves of *Combretum albidum* G. Don. Anc. Sci. Life.

[97] Harborne J. (1973). Phytochemical Methods. A Guide to Modern Techniques of Plant Analysis.

[98] Trease G., Evans W. (1989). Pharmacognosy.

[99] Sofowora A. (1996). Research on medicinal plants and traditional medicine in Africa. J. Altern. Complement. Med..

